# The association between cognitive inhibitory control and language performance in post-stroke aphasia: a systematic review and meta-analysis

**DOI:** 10.3389/fpsyg.2026.1846757

**Published:** 2026-05-26

**Authors:** Ming-xi Xu, Ying-ying Yang, Xia He, Xia-lian Huang, Xiao-qin Yun, Xiao-xue Zeng, Feng-le Mao, Yan-qiu Wang, Fu-li Qin

**Affiliations:** 1 School of Health Preservation and Rehabilitation, Chengdu University of Traditional Chinese Medicine, Chengdu, China; 2 Sichuan Provincial Bayi Rehabilitation Center (Sichuan Provincial Rehabilitation Hospital), Chengdu, China

**Keywords:** correlation coefficient, inhibitory control, language performance, meta-analysis, post-stroke aphasia, systematic review

## Abstract

**Background:**

Inhibitory control, a core component of executive function, may play an important role in language processing in individuals with post-stroke aphasia. However, the evidence on its association with language performance has not been systematically synthesized. This systematic review and meta-analysis aimed to examine the relationship between inhibitory control and language performance in post-stroke aphasia.

**Methods:**

This meta-analysis was conducted in accordance with the Preferred Reporting Items for Systematic Reviews and Meta-Analyses (PRISMA) guidelines. A systematic search of six databases was conducted from database inception to February 1, 2026, for studies examining the association between inhibitory control and language performance. Two independent researchers conducted literature screening, data extraction, and quality evaluation using the Newcastle-Ottawa Scale. The meta-analysis was performed using Comprehensive Meta-Analysis software, version 3 (CMA 3.0), with correlation coefficient (*r*) as the effect size. Effect sizes were transformed to Fisher’s z and pooled using the inverse-variance method under a fixed-effects model. Heterogeneity was assessed using the Q-test and *I^2^* statistic. Subgroup analyses were conducted according to the type of inhibitory control and language domain. Leave-one-out sensitivity analysis and Egger’s test were used to assess robustness and publication bias.

**Results:**

A total of 12 studies involving 357 individuals with post-stroke aphasia were included. The pooled analysis showed a significant positive correlation between inhibitory control and language performance (*r* = 0.339, 95% CI [0.239, 0.432], *p* < 0.001), with no observed heterogeneity (*I^2^* = 0.0%). Subgroup analysis showed that interference control was significantly associated with global language function, expressive language performance, and receptive language performance, whereas response inhibition was significantly associated only with global language function. However, between-subgroup differences were not statistically significant. Sensitivity analyses indicated robust results, and no significant publication bias was detected.

**Conclusion:**

Inhibitory control is significantly associated with language performance in post-stroke aphasia. Different components of inhibitory control may show distinct patterns of association across language domains, with interference control relating to a broader range of language abilities. These findings suggest that inhibitory control may be an important factor in the assessment and rehabilitation of post-stroke aphasia.

**Systematic review registration:**

https://www.crd.york.ac.uk/prospero/, identifier CRD420261303657.

## 1 Introduction

Stroke is one of the leading causes of death and disability worldwide, with significant negative impacts on the economy and people’s quality of life (Kuriakose and Xiao, 2020). Post-stroke aphasia (PSA) is a common acquired language disorder following stroke (Burton et al., 2023), characterized by impaired language performance across multiple domains, including spoken expression, auditory comprehension, naming, repetition, reading, and writing (Burgio and Basso, 1997; Flowers et al., 2016). It has been estimated that over 10 million people worldwide experience stroke each year, with more than one-third of them developing aphasia, significantly impacting patients’ daily communication and social participation (Lazar and Boehme, 2017). In addition to language impairment, patients with post-stroke aphasia often experience varying degrees of cognitive impairment due to impaired cognitive processes, leading to poor functional recovery, adverse outcomes, and reduced quality of life (Ardila and Rubio-Bruno, 2018; Lee and Pyun, 2014; Wang et al., 2019). Among these cognitive processes, inhibitory control is a core component of executive function (Diamond, 2013). It is involved in the suppression of irrelevant information, the regulation of competing activation, and the selection of target responses (Hussey and Novick, 2012; Kuzmina and Weekes, 2017), and thus may be closely related to language performance. Importantly, inhibitory control is not a unitary construct, but includes partially separable components such as interference control, which refers to suppressing distracting or competing information, and response inhibition, which refers to withholding or stopping a prepotent response (Miyake et al., 2000; Nigg, 2000; Friedman and Miyake, 2004). Previous study has found that among patients with aphasia, those with better inhibitory control are more likely to exhibit generalization effects after naming therapy, and the successful naming of generalized items may largely depend on the inhibition of competing non-target lexical items (Yeung et al., 2009). Furthermore, existing studies have indicated that aphasia patients may still retain some linguistic knowledge and representations, but their language performance is limited by their ability to organize and regulate information during language processing (Hula and McNeil, 2008). The dynamic activation and inhibition of language-related information suggest that inhibitory control may be closely involved in language processing and performance. Therefore, clarifying its relationship with language performance is of particular significance.

However, existing research has largely focused on the relationship between executive function in a broad sense and aphasia. Inhibitory control is typically included in overall analyses as a component of executive function, with few studies specifically investigating its association with language performance. Previous studies suggest that inhibitory control may play a role in language processing (Jefferies et al., 2009; Novick et al., 2010), but direct evidence in patients with post-stroke aphasia remains limited. Meanwhile, the relationships between different inhibitory control components and different dimensions of language performance still need to be further clarified.

Furthermore, there remains a lack of systematic synthesis and quantitative analysis of the relationship between inhibitory control and language performance in post-stroke aphasia. Therefore, this study aims to investigate the association between inhibitory control and language performance in patients with post-stroke aphasia. Particular attention was given to the associations between different inhibitory control components and language performance, in order to further elucidate the cognitive-linguistic mechanisms underlying PSA and to inform clinical assessment and rehabilitation.

## 2 Methods

This meta-analysis adheres to the PRISMA (Preferred Reporting Items for Systematic Reviews and Meta-Analyses) 2020 statement (Page et al., 2021). We conducted the systematic review based on an *a priori* protocol registered in the International Prospective Register of Systematic Reviews (PROSPERO; identifier number CRD420261303657).

### 2.1 Literature search

We conducted a systematic literature search of PubMed, Embase, the Cochrane Library, Web of Science, Chinese National Knowledge Infrastructure (CNKI), and Wanfang databases, covering the period from the inception of each database to February 1, 2026. We focused on studies assessing the relationship between inhibitory control and language performance in individuals with post-stroke aphasia. In addition, manual searches of reference lists from existing studies were conducted. The search strategy combined subject headings, where available, and free-text terms related to stroke (e.g., stroke, post-stroke, cerebrovascular accident [CVA], cerebral infarction), aphasia/language impairment (e.g., aphasia, speech disorder, anomia, language disorder), and inhibitory control/executive control (e.g., inhibitory control, inhibition, executive function, response inhibition, cognitive control, attention, Stroop, Flanker, Simon task, Go/No-Go, Stop-signal, and Hayling). No language restrictions were applied. Database-specific full search strings are provided in Supplementary material 1. Two investigators (MXX and YYY) independently searched the reference lists of all identified articles and gray literature for potentially eligible studies. EndNote 21.4 (Clarivate, Philadelphia, PA, USA) was used to organize the search results and remove duplicates.

### 2.2 Identification of eligible studies

Studies were included if they met the following criteria: (1) reported original quantitative data from individual-level studies, including observational studies and baseline data from intervention studies; (2) included adult participants aged ≥18 years with aphasia secondary to ischemic or hemorrhagic stroke; (3) included participants with post-stroke aphasia at any stage of stroke recovery (acute, subacute, or chronic); (4) included participants with any aphasia subtype, including studies with mixed aphasia presentations; (5) assessed inhibitory control using at least one validated behavioral paradigm (e.g., Flanker/ANT, Stroop, Go/No-Go, Stop-signal, Simon, or comparable tasks); and (6) examined the association between inhibitory control and language outcomes (e.g., naming, repetition, comprehension, fluency, or overall aphasia severity) and reported Pearson’s correlation coefficient (*r*) or Spearman’s rank correlation coefficient (*ρ*) to calculate effect sizes. For intervention studies, only pre-intervention baseline data were extracted to quantify the cross-sectional association between inhibitory control and language performance. Studies including a small proportion of non-stroke etiologies that could not be separated were retained and examined in sensitivity analyses. Studies were excluded if they met any of the following criteria: (1) the full text was unavailable or the raw data were missing; (2) non-original publications, including meta-analyses, reviews, editorials/letters, protocols, conference abstracts, and case reports/case series; (3) studies without sufficient data to compute effect sizes even after attempting to contact the authors.

### 2.3 Data extraction

Two investigators (MXX and YYY) independently extracted data from all eligible studies. Disagreements were settled through consultation with a third investigator (XH). Extracted data included study and participant characteristics (sample size, sex distribution, time post onset, and mean age), inhibitory control measures, and language measures together with the specific outcome indices and subdomains reported. Different studies may report different subtest results or use different derived indices even when the same scale is applied. Accordingly, effect sizes were not directly pooled by scale name, but were classified and selected on the basis of predefined outcome categories.

Inhibitory control tasks were categorized into interference control and response inhibition. Interference control tasks included the Flanker Task (Mullane et al., 2009), the Stroop task (Scott and Wilshire, 2010) and a Flanker-like Nonverbal Control Task (Gray, 2020). Response inhibition tasks included the Go/No-Go Task (Cragg and Nation, 2008) and the Stop-Signal Task (Verbruggen and Logan, 2008). For tests or tools that assessed broader executive functions, only the components or subtest scores related to inhibitory control were extracted. Measures reflecting other non-inhibitory dimensions were excluded from extraction and pooled analysis. Language outcomes were derived from both comprehensive aphasia batteries and domain-specific language measures, and were classified according to the reported outcome indices and the language domain assessed. For subsequent analyses, language measures were grouped into three domains: global language function, receptive language performance, and expressive language performance. Global language function comprised composite total scores or severity indices reflecting overall language ability or aphasia severity, typically derived from comprehensive aphasia batteries. Receptive language comprised measures of input-related processing, such as auditory or reading comprehension. Expressive language comprised measures of output-related processing, including naming, spoken production, lexical retrieval, and repetition.

For each included study, detailed information was extracted to ensure transparency and traceability. The extracted outcomes included the reported direction and significance of associations, together with Pearson correlation coefficients where available. Detailed information on the extracted inhibitory control and language measures, derivation methods, effect-direction harmonization rules, and variable mapping is provided in Supplementary material 2.

### 2.4 Quality assessment

Two reviewers (MXX and YYY) independently assessed the methodological quality of the included studies using the Newcastle-Ottawa Scale (NOS) (Stang, 2010). The NOS evaluates three domains: selection, comparability, and exposure/outcome assessment. The scale consists of eight items scored on a 9-point scale, with a score of 6 or higher indicating a high-quality study. Any disagreements were resolved through discussion with a third investigator (XH).

### 2.5 Statistical analysis

Meta-analyses were conducted using Comprehensive Meta-Analysis software, version 3. Although the included studies varied in design, only association data measured at a single time point were extracted for the meta-analysis; for intervention studies, only pre-intervention data were used. Accordingly, the effect size of interest was the association between inhibitory control and language performance within each sample, rather than a pre–post change effect. Pearson’s correlation coefficient (*r*) was used as the primary effect size index (Wang and Li, 2022). Eleven studies reported Pearson’s *r*, whereas one study (Kendrick et al., 2019) reported Spearman’s *ρ*. Given that only one study reported *ρ* and its sample size was small (*n* = 10), Spearman’s *ρ* was treated as an approximation to Pearson’s *r* for the purpose of synthesis. Where sufficient data were available, subgroup analyses of inhibitory control and language performance were conducted within these subdomains. When a study reported multiple effect sizes, a prespecified selection and aggregation strategy was used to avoid double-counting correlated outcomes. For the overall analysis, one representative effect size was derived from each study to estimate the general association between inhibitory control and language performance. Correlations based on overall language scores, such as the Aphasia Quotient of the Western Aphasia Battery (WAB-AQ), were preferentially extracted. If no overall language score was available, the correlation coefficients for relevant component measures within the same study were transformed into Fisher’s z values using the formula $z_{i}=0.5\ln[(1+r_{i})/(1-r_{i})]$ (Borenstein, 2009). The variance of each z value was calculated as $V_{i}=1/(n_{i}-3)$ , and the corresponding inverse-variance weight was defined as $w_{i}=1/V_{i}$ . Because most primary studies did not report the intercorrelations required to model dependence among multiple outcomes within the same sample, these z values were combined using inverse-variance weighting to derive a single study-level effect size. The pooled effect size was converted back to a Pearson correlation coefficient using the inverse Fisher’s z transformation: $\;r=(e^{2z}-1)/(e^{2z}+1)$ . For subgroup analyses, effect sizes were extracted according to prespecified subgroup categories. If a study reported distinct effect sizes for different subgroups, these were entered into the corresponding subgroup analyses. Within each subgroup, multiple effect sizes from the same study were combined into a single subgroup-specific study-level effect size using the procedure described above. Because one study could contribute to multiple subgroups, only subgroup-specific pooled estimates were reported, and no overall pooled estimate across subgroups was further calculated.

Between-study heterogeneity was assessed using Cochran’s Q test (*α* = 0.10) and quantified using the *I^2^* statistic; values of approximately 25, 50, and 75% were interpreted as low, moderate, and high heterogeneity, respectively. Forest plots were generated with 95% confidence intervals. Effect sizes were interpreted using conventional thresholds for correlation coefficients, with *r* values of 0.10, 0.30, and 0.50 indicating small, moderate, and large effects, respectively. A fixed-effects model was adopted when *I^2^* ≤ 50%, whereas a random-effects model was used when *I^2^* > 50% (Higgins et al., 2003; Stang, 2010). Figures were formatted and enhanced using R 4.4.3.

To ensure consistent interpretation of effect direction, correlations were coded such that a positive value always indicated that better inhibitory control was associated with better language performance. Thus, when inhibitory control was indexed by measures in which lower scores reflected better performance (e.g., reaction time on Stroop-type tasks), the sign of the correlation was reversed when it is necessary before analysis.

### 2.6 Subgroup analysis

Prespecified subgroup analyses were conducted by inhibitory control types (interference control and response inhibition) and language domains (global language function, expressive language performance, and receptive language performance). Effect sizes were pooled using fixed-effects models, and pooled estimates with 95% confidence intervals were calculated for each subgroup. Subgroup differences were tested with the Q-between statistic. To avoid double-counting in the overall meta-analysis, only one effect size per study was included according to a predefined prioritization strategy.

### 2.7 Sensitivity analysis

The present study performed a leave-one-out sensitivity analysis to assess the influence of individual studies on the overall results. In this procedure, the meta-analysis was repeated after sequentially removing one study at a time to evaluate the influence of each individual study on the pooled effect size (Viechtbauer and Cheung, 2010). This analysis was used to examine the robustness of the overall findings and to determine whether any single study had a disproportionate impact on the magnitude or statistical significance of the pooled estimate. If the pooled effect size remained stable across iterations, the results were considered robust.

### 2.8 Publication bias

When 10 or more studies were included, we used CMA 3.0 to perform Egger’s regression test and assessed publication bias using funnel plots (Egger et al., 1997).

## 3 Results

### 3.1 Literature search and study characteristics

**Figure 1:**
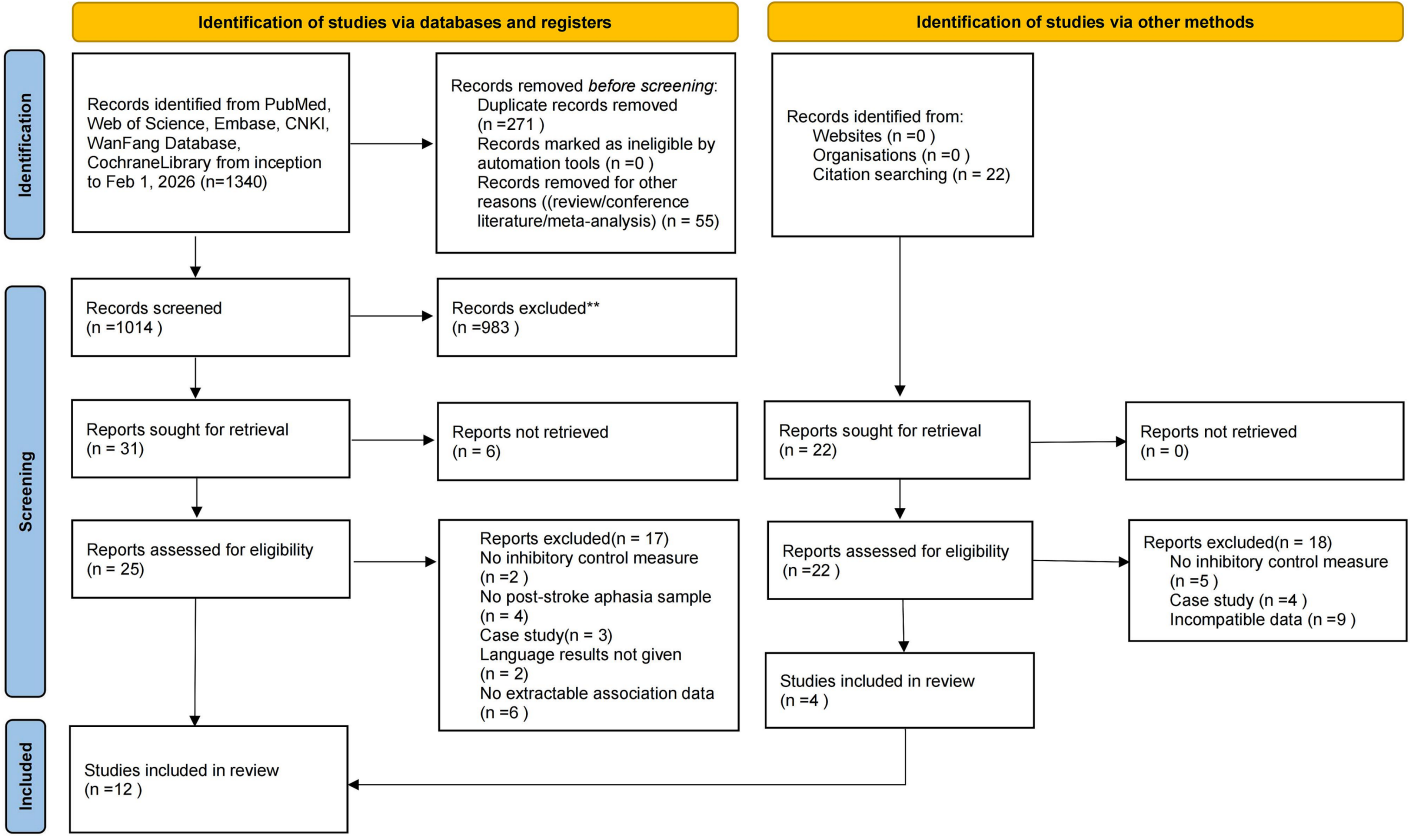
PRISMA flow chart of literature searching and screening.

The database search identified 1,340 records, and an additional 22 records were identified through citation searching. After removal of 271 duplicates and 55 clearly ineligible non-original records (e.g., reviews, conference papers, and meta-analyses), 1,014 titles/abstracts were screened. A total of 31 reports from the database search and 22 reports from citation searching were sought for retrieval. Following assessment against the inclusion and exclusion criteria, a total of 12 studies (Allen et al., 2011; DeDe et al., 2025; Faroqi-Shah and Gehman, 2021; Gray, 2020; Kendrick et al., 2019; Lee et al., 2020; Lenz and LaCroix, 2025; Meier et al., 2022a; Musso et al., 2022; Schumacher et al., 2022; Simic et al., 2020; Votruba et al., 2013) were included in this review. The PRISMA 2020 flow diagram (Figure 1) illustrates the process of study selection and the reasons for excluding studies (Page et al., 2021). The main characteristics of the included studies are summarized in Table 1. All included studies reported data on the association between inhibitory control and language performance. Of the 12 studies, nine were cross-sectional, one was a prospective cohort study, one was a non-randomized single-group pre–post intervention study, and one was a longitudinal intervention study. Sample sizes ranged from 10 to 114, with a total of 357 participants. Participants were predominantly aged 55–65 years, and the proportion of male participants was generally high (43.8–90%); only one study did not report sex distribution. Stroke chronicity varied considerably across studies, ranging from the acute stage (0.18 months, approximately 5.3 days) to the very chronic stage (up to 103.1 months), although one study did not report time post onset. Most studies included participants with mixed aphasia presentations rather than restricting recruitment to a single aphasia subtype. Two studies also included a very small proportion of individuals with traumatic brain injury. With regard to language background, all studies included monolingual participants except one involving bilingual individuals with aphasia. The median Newcastle–Ottawa Scale score across the 12 studies was 8 (range, 6–9; Table 2). Quality scores ranged from 6 to 9 for cross-sectional studies and from 7 to 9 for the remaining studies, with no study rated as low quality. Most studies showed adequate quality overall, although some variability was observed in the selection and comparability domains, particularly with respect to adjustment for confounding variables. Nevertheless, the findings should be interpreted with caution, as most included studies were cross-sectional and differed in sample characteristics, task paradigms, language measures, and confounder adjustment. These factors may have reduced comparability across studies, while the predominance of cross-sectional designs limits causal inference.

**Table 1:** Characterization of the studies included in the systematic review.

Author (year)	Sample	Mean age (SD)	% Male	TPO (mo)	Inhibition task	Inhibition domain	Language measure	Language domain	*r*
Meier et al. (2022a)	14	62.29 (9.80)	57.1%	0.18^†^	Flanker	IC	WAB-R; BNT	Glo; Exp	0.379*
Kendrick et al. (2019)	10	64.27 (8.12)	70%	88.8	Flanker, Stroop	IC	CAT; CCT	Exp, Rec	0.396*
Lenz and LaCroix (2025)	58	59.47 (10.02)	58.6%	83.85	ANT	IC	WAB-R	Glo	0.480
Schumacher et al. (2022)	32	61.6 (11.4)	59.4%	78.0	ECwD, TAP	IC&RI	CAT	Glo	0.341*
Lee et al. (2020)	114	56.5 (12.3)	54.4%	35.1	CPT-II	RI	WAB-R	Glo	0.275
Simic et al. (2020)	10	55.5(15.04)	90%	18.10	Mixed	IC&RI	BNT	Exp	0.444*
Votruba et al. (2013)	50	56.8 (15.2)	56%	43.6	SST	RI	BNT, BDAE	Exp, Rec	0.328*
Faroqi-Shah and Gehman (2021)	14	63.7 (11.4)	50%	NR	Stroop	IC	WAB-R	Glo	0.230
Allen et al. (2011)	20^^^	62.6	NR	9.63	Mixed	IC&RI	SPC	Glo	0.150
Musso et al. (2022)	10	58.4 (11.7)	90%	29.9	Go/NoGo	RI	AAT	Glo	0.289*
DeDe et al. (2025)	13	59.9 (11.2)	43.8%	103.1	Stroop	IC	BDAE, PNT	Glo; Exp	−0.024*
Gray (2020)	12^^^	51 (11.0)	75%	77.4	NCT	IC	LCT	Exp	0.280

AAT = Aachen Aphasia Test; ANT = Attention Network Test; BNT = Boston Naming Test; BDAE = Boston Diagnostic Aphasia Examination; CAT = Comprehensive Aphasia Test; CCT = Camel and Cactus Test; CPT-II = Conners’ Continuous Performance Test-II; ECwD = Elevator Counting with Distraction; Flanker = Flanker Inhibitory Control; Go/NoGo = Go/NoGo-task; LCT = Language Control Task; NCT = Nonverbal Control Task; PNT = Philadelphia Naming Test; SPC = Semantic Processing Composite; SST = Stop Signal Task; Stroop = Stroop inhibition tasks; TAP = Test of Attentional Performance; TPO = Time Post Onset; WAB-R = Western Aphasia Battery-Revised. Domains: Exp = expressive language performance; IC = interference control; Rec = receptive language performance; RI = response inhibition; Glo = global language function; NR = Not Reported.

*The correlation (*r*) value is the mean correlation calculated using Fisher’s Z-to-r transformation.

^†^Original TPO value reported as 5.28 days, converted to months for consistency.

^^^Sample includes individuals with Traumatic Brain Injury (Allen: 5%; Gray: 17%).

**Table 2:** Risk of bias assessment according to the Newcastle-Ottawa Scale.

Reference	Study design	Selection	Comparability	Exposure/outcome	Total
Meier et al. (2022a)	Cross-sectional	●●●	●	●●●	7
Kendrick et al. (2019)	Cross-sectional	●●●	●	●●●	7
Lenz and LaCroix (2025)	Cross-sectional	●●●●	●●	●●	8
Schumacher et al. (2022)	Cross-sectional	●●●●	●●	●●●	9
Lee et al. (2020)	Cross-sectional	●●●●	●●	●●●	9
Simic et al. (2020)	Longitudinal intervention	●●●	●	●●●	7
Votruba et al. (2013)	Prospective cohort	●●●●	●●	●●●	9
Faroqi-Shah and Gehman (2021)	Cross-sectional	●●●	●●	●●●	8
Allen et al. (2011)	Cross-sectional	●●	●●	●●	6
Musso et al. (2022)	Proof-of-concept	●●●	●●	●●●	8
DeDe et al. (2025)	Cross-sectional	●●	●●	●●●	7
Gray (2020)	Cross-sectional	●●●	●●	●●●	8

### 3.2 Overall meta-analysis

#### 3.2.1 The relationship between inhibitory control and language performance

**Figure 2:**
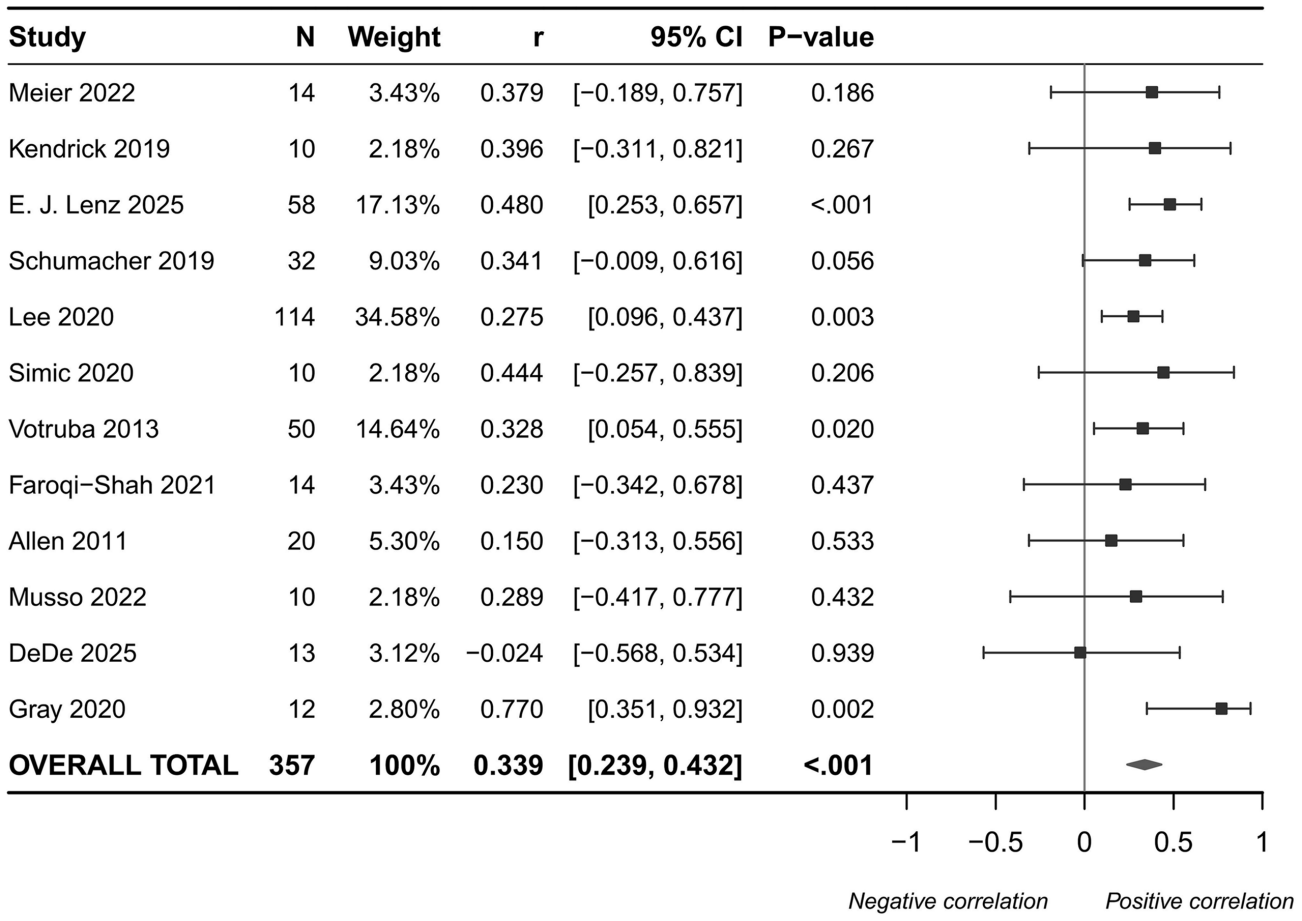
Forest plot of the association between inhibitory control and language performance.

A total of 12 studies (total sample size *N* = 357) were included in the overall correlation analysis. Given the negligible heterogeneity across studies (*Q* = 8.617, *p* = 0.657, *I^2^* = 0.0%), a fixed-effects model was used. The pooled effect size, back-transformed to Pearson’s r for interpretation, indicated a moderate positive association between inhibitory control and language performance (*r* = 0.339, 95% CI [0.239, 0.432]; *p* < 0.001) (Figure 2). These results indicate that post-stroke aphasia patients with higher inhibitory control scores demonstrate better language performance. The direction of the association was generally consistent across studies: 11 studies reported positive correlations, whereas one study (DeDe et al., 2025) reported a slight negative correlation (*r* = −0.024). The effect sizes across the studies ranged from −0.024 to 0.77. The largest contributions came from Lee et al. (2020) and Lenz and LaCroix (2025), which together accounted for more than half of the total weight.

### 3.3 Subgroup analysis

Prespecified subgroup analyses were conducted to examine whether the association between inhibitory control and language performance differed across inhibitory control domains and language domains. Effect sizes were stratified by inhibitory control domains (interference control and response inhibition) and language domains (global language function, expressive language performance, and receptive language performance).

#### 3.3.1 Interference control and language performance

**Figure 3:**
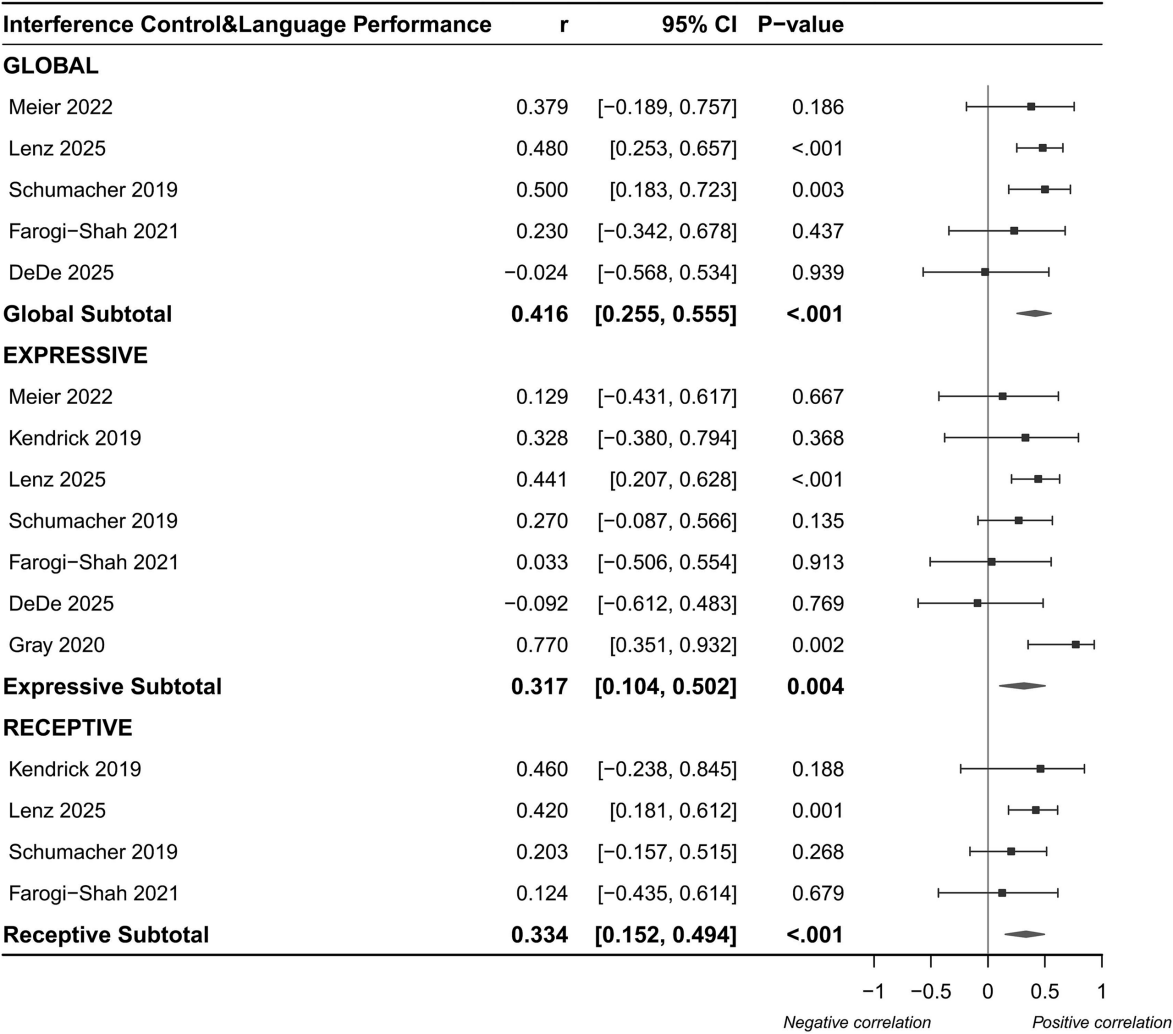
Forest plot of the association between interference control and language performance.

Seven studies contributed data on the association between interference control and language performance. Using a fixed-effects model, the pooled results showed that interference control was significantly positively associated with all three language domains: global language function (*r* = 0.416; 95% CI [0.255, 0.555]; *p* < 0.001), expressive language performance (*r* = 0.317; 95% CI [0.104, 0.502]; *p* = 0.004), and receptive language performance (*r* = 0.334; 95% CI [0.152, 0.494]; *p* < 0.001) (Figure 3). Among these, the strongest pooled association was observed for global language function.

#### 3.3.2 Response inhibition and language performance

**Figure 4:**
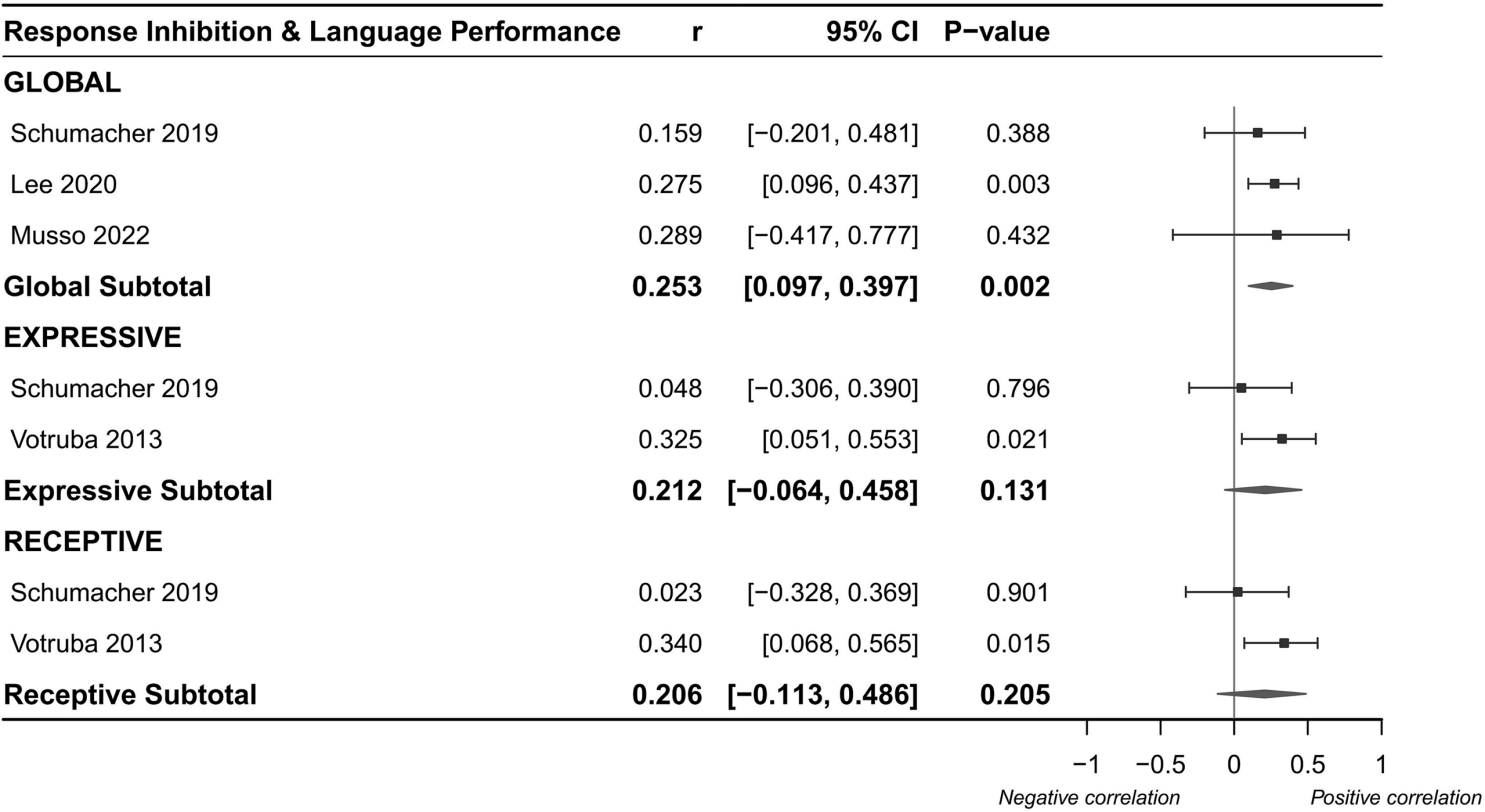
Forest plot of the association between response inhibition and language performance.

Four studies contributed data on the association between response inhibition and language performance. A fixed-effects meta-analysis showed that response inhibition was significantly positively associated with global language function (*r* = 0.253, 95% CI [0.097, 0.397]; *p* = 0.002), whereas its associations with expressive language performance (*r* = 0.212; 95% CI [−0.064, 0.458]; *p* = 0.131) and receptive language performance (*r* = 0.206; 95% CI [−0.113, 0.486]; *p* = 0.205) were not statistically significant (Figure 4).

#### 3.3.3 Between-subgroup difference test: interference control and response inhibition

When pooled across language domains, the correlation coefficient was numerically higher for interference control than for response inhibition (*r* = 0.367; 95% CI [0.211, 0.505] and *r* = 0.255; 95% CI [0.120, 0.382]). However, the between-subgroup difference was not statistically significant ( $Q_{\text{between}}=1.204$ ; df=1 ; *p* = 0.272). The correlation plots are presented in Supplementary material 3.

### 3.4 Sensitivity analysis

**Figure 5:**
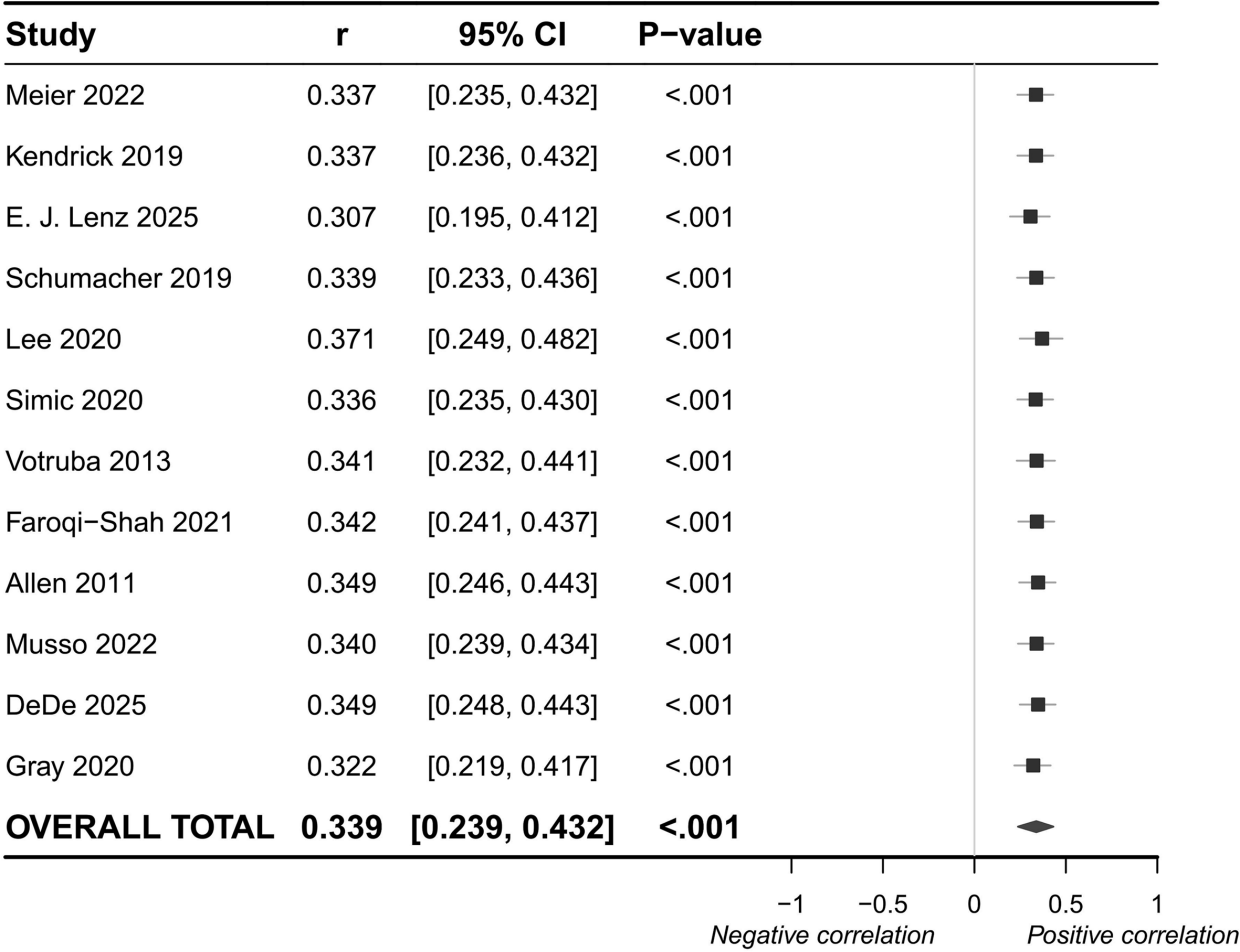
Sensitivity analysis of inhibitory control and language performance.

The leave-one-out sensitivity analysis indicated that the pooled correlation coefficient was robust to the sequential exclusion of individual studies, ranging from 0.307 to 0.371, and all pooled estimates remained significantly positive (all *p* < 0.001). In addition, none of the corresponding 95% confidence intervals crossed zero, with lower and upper bounds ranging from 0.195 to 0.482, respectively (Figure 5). With all studies included, the overall pooled effect size was *r* = 0.339 (95% CI [0.239, 0.432]). To sum up, these results suggest that the overall finding was stable and not unduly influenced by any single study, including the study that reported Spearman’s *ρ*.

### 3.5 Publication bias

**Figure 6:**
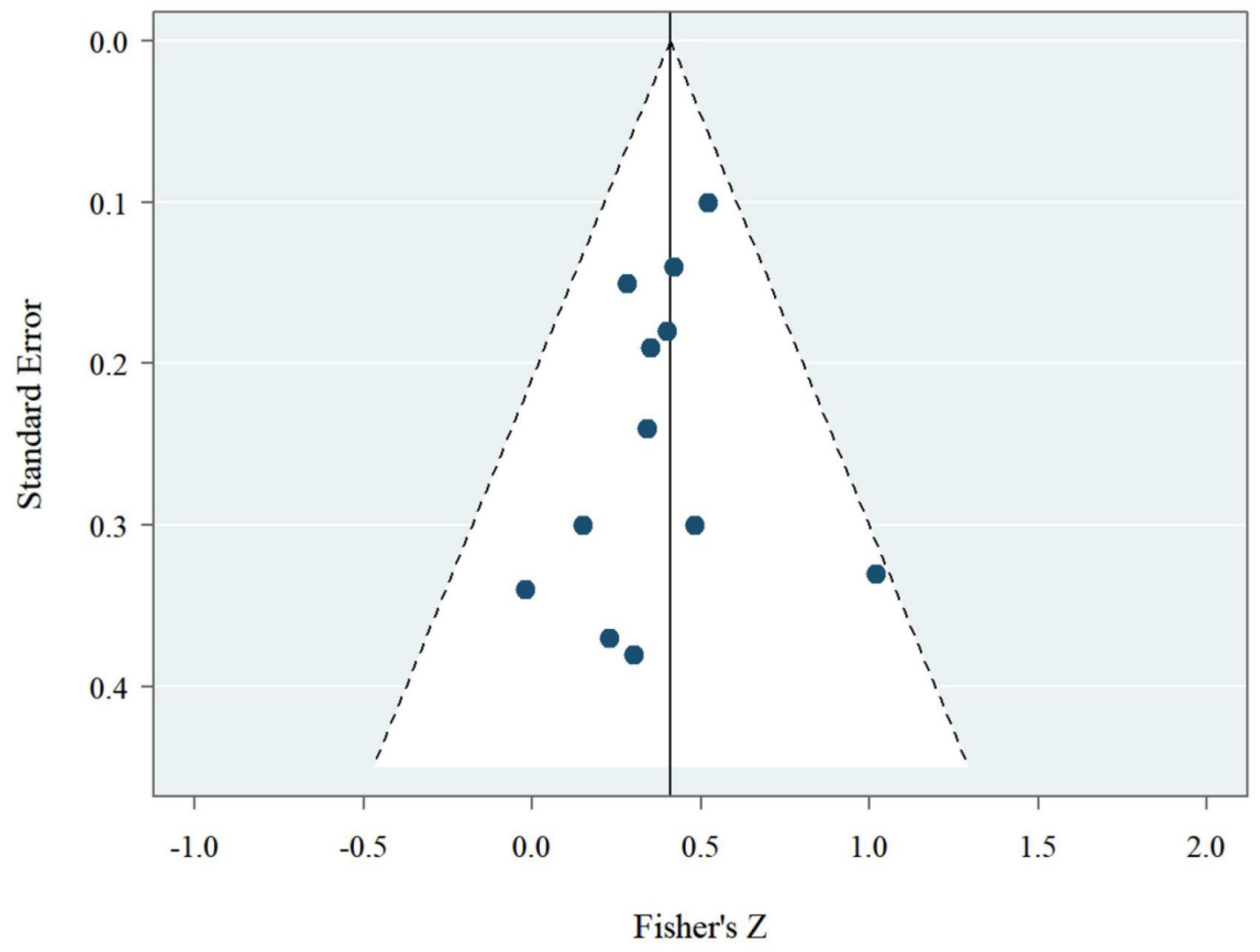
Funnel plots of inhibitory control and language performance.

Publication bias was assessed for the association between inhibitory control and language performance. The funnel plot was largely symmetrical (Figure 6), and Egger’s test (*p* = 0.718) indicated no significant bias.

## 4 Discussion

The present study aimed to determine whether inhibitory control is associated with language performance in individuals with post-stroke aphasia and to further examine whether this association varies across inhibitory control subcomponents and language domains. The meta-analytic results revealed a significant moderate positive association between overall inhibitory control and language performance, indicating that individuals with better inhibitory control tend to exhibit better language performance. Importantly, this association should be interpreted as correlational rather than causal, given that most of the included studies used cross-sectional designs. The absence of significant heterogeneity suggests that the overall association was relatively consistent across studies. Sensitivity analyses further indicated that the pooled effect was not driven by any single study, supporting the robustness of the findings. Although one study (DeDe et al., 2025) showed a very small negative association that differed from the overall pattern, this discrepancy may be related more to task design and sample characteristics than to instability of the overall effect. Subgroup analyses further showed positive associations across both inhibitory control subtypes and language domains. At a descriptive level, interference control appeared to be associated with a broader range of language domains, whereas response inhibition was more selectively related to global language function. However, because between-subgroup differences did not reach statistical significance, these patterns should be interpreted cautiously as preliminary trends rather than definitive evidence of domain or component-specific effects.

Inhibitory control refers to the ability to suppress irrelevant information, dominant responses, and internal interference in the service of goal-directed behavior (Diamond, 2013). This function is highly relevant to language processing because language requires both the activation of target representations and the suppression of competing lexical, semantic, and syntactic information (Novick et al., 2005; Zhang et al., 2025). During ambiguous word processing and sentence comprehension, inhibitory control supports the suppression of non-target meanings, the inhibition of initially inappropriate interpretations, and the revision of those interpretations when necessary (Hussey and Novick, 2012). In language production, inhibitory control supports lexical selection by suppressing competing candidates during word retrieval (Schnur et al., 2006). Together, evidence from comprehension and production suggests that inhibitory control contributes to language performance by regulating competition among concurrently activated representations. More generally, inhibitory control is associated with the ability to select context-appropriate word meanings and suppress inappropriate alternatives (Jefferies et al., 2009; Novick et al., 2010). This role may be especially important in post-stroke aphasia, because, in addition to damage to the language system itself, many patients also show executive control impairments, including deficits in inhibitory control. Such deficits may make it more difficult to suppress irrelevant stimuli, competing lexical items, or erroneous interpretations during language processing, thereby contributing to poorer comprehension and production (Kendrick et al., 2019; Kuzmina and Weekes, 2017; Meier et al., 2022b).

However, while inhibitory control may help explain the overall language association, the non-uniform pattern across language measures suggests that subgroup differences may instead reflect the multidimensional nature of both inhibitory control and language performance. Interference control and response inhibition may place different demands on language processing. Interference control is primarily involved in selecting targets under competitive conditions and suppressing irrelevant inputs (Friedman and Miyake, 2004), whereas response inhibition mainly refers to stopping or blocking the prepared dominant reaction, and is more closely related to output-level control (Friedman and Miyake, 2004; Nigg, 2000).

Language performance is likewise multifaceted. Expressive language engages lexical retrieval, phonological-articulatory planning, and sentence formulation, whereas receptive language depends on mapping speech input onto semantic and syntactic structure and integrating these representations online (Hagoort, 2005; Indefrey, 2011). Global language function, by contrast, reflects overall language ability across multiple domains (Landrigan et al., 2021). Against this background, distinct inhibitory control components may show different patterns of association with language performance. Interference control, which operates at earlier stages of representational selection, may support language performance by facilitating the selection of relevant information and suppressing competing lexical or syntactic representations (Friedman and Miyake, 2004; Jefferies et al., 2008; Ye and Zhou, 2009). Consistent with this account, lexical selection is more vulnerable under semantic interference, and syntactic ambiguity resolution recruits conflict-sensitive control mechanisms, suggesting that efficient interference control contributes to better language performance (January et al., 2009; Schnur et al., 2006). From a neural perspective, interference control in language has been linked to the left inferior frontal/ventrolateral prefrontal cortex, a region engaged when selection demands increase and multiple semantic representations compete for access (Badre and Wagner, 2007; Thompson-Schill et al., 1997). Response inhibition, by contrast, is primarily involved in suppressing prepotent or ongoing motor responses at the behavioral output stage (e.g., action withholding and action cancellation), rather than directly resolving competition among lexical or syntactic representations (Swick et al., 2011; Verbruggen et al., 2019; Zhang et al., 2017). Although response inhibition often co-occurs with performance monitoring, error monitoring is a related but partially dissociable control process (Ullsperger et al., 2014). It has been most consistently associated with a fronto-basal-ganglia stopping network involving the right inferior frontal cortex and the presupplementary motor area (pre-SMA), rather than being primarily implicated in lexical or syntactic selection(Aron et al., 2014; Zhang et al., 2017). Therefore, interference control may be relevant across a broader range of language domains, whereas response inhibition may be more evident in composite measures of global language function because composite indices aggregate performance across multiple subtests and may be more sensitive to domain-general output-control demands (Meier et al., 2022a). However, because between-subgroup comparisons were not statistically significant, this interpretation should be regarded as tentative and descriptive rather than as evidence for definitive category-specific differences.

In recent years, research on post-stroke aphasia has largely treated executive function as a broad construct, but an increasing number of studies have incorporated inhibition-related tasks and measures when examining their relationship with language performance (Lenz and LaCroix, 2025; Meier et al., 2022a; Musso et al., 2022). Although inhibitory control was not always the primary focus, these studies converge in suggesting that better inhibitory control is associated with better language performance in post-stroke aphasia, consistent with the present findings. It should also be noted that previous reviews have examined the relationship between language performance or recovery and executive function from a broad perspective in post-stroke aphasia (Simic et al., 2019), primary progressive aphasia (Coemans et al., 2022; Thomsen et al., 2024), and bilingual aphasia (Dash and Kar, 2014). However, these reviews have mainly discussed executive function at a general level, and there is still a lack of systematic synthesis focusing specifically on inhibitory control. Given that executive function is characterized by both unity and diversity, with different subcomponents being interrelated yet separable and contributing differently to performance on complex tasks (Miyake et al., 2000), examining executive function only at the overall level may not be sufficient to reveal which specific components are more closely related to language performance. Accordingly, the present study focused specifically on inhibitory control and quantitatively synthesized its relationship with language performance in aphasia, providing more specific evidence for this association.

However, some limitations should also be acknowledged in our study. Firstly, the number of included studies and the overall sample size were relatively limited, especially in the subgroup analyses, where some inhibitory control components and language domains were represented by only a few studies. Therefore, these findings require further verification in larger samples. Secondly, although no significant heterogeneity was observed in the overall analysis, there were still differences across studies in inhibitory control task paradigms, language assessment tools, and sample characteristics, which may have affected the comparability of effect sizes. Additionally, the classification of language performance in this study was relatively broad, making it difficult to further examine the correspondence between more specific language representations processes and different inhibitory control components. Future research should adopt more refined classifications to further investigate the differentiated associations between various subcomponents of executive function and language performance. Finally, because most of the included studies employed cross-sectional designs, the findings mainly reflect an association between inhibitory control and language performance rather than a causal relationship. Despite the above limitations, this meta-analysis provides new quantitative evidence on the relationship between inhibitory control and language performance in post-stroke aphasia. To our knowledge, this is the first meta-analysis to systematically quantify this association. The findings suggest that inhibitory control may be a relevant factor associated with language performance in post-stroke aphasia. Clinically, assessing inhibitory control together with language performance may help characterize patients’ cognitive-linguistic profiles more comprehensively. In rehabilitation, the observed association raises the possibility that inhibitory control training could be explored as an adjunct to language therapy, particularly for language tasks involving lexical competition, semantic ambiguity, or suppression of irrelevant responses. However, the present findings do not demonstrate that inhibitory control training improves language performance. Future longitudinal and intervention studies are needed to determine whether improving inhibitory control can causally contribute to better language performance.

## 5 Conclusion

In conclusion, this review found a significant positive association between inhibitory control and language performance in individuals with post-stroke aphasia. This result suggests that inhibitory control may be an important cognitive factor related to language performance in post-stroke aphasia. The findings support considering inhibitory control in cognitive-linguistic assessment, treatment planning, and rehabilitation monitoring. Integrating inhibitory control into clinical practice may facilitate more individualized rehabilitation strategies. However, because current evidence is limited by small samples, methodological variability, and predominantly cross-sectional designs, larger prospective studies are needed to confirm and refine these conclusions.

## Data Availability

The original contributions presented in the study are included in the article/Supplementary material, further inquiries can be directed to the corresponding author.
