# Supplementary material for: The association between cognitive inhibitory control and language performance in post-stroke aphasia: a systematic review and meta-analysis
Source: Front Psychol. 2026 May 26;17:1846757. doi: 10.3389/fpsyg.2026.1846757 (PMC13246680; doi:10.3389/fpsyg.2026.1846757)
Supplement: Supplementary file 1 [file Supplementary_File_1.docx]

Search strategy

Searches were conducted in PubMed, Embase, the Cochrane Library, Web of Science, CNKI, and Wanfang Data from database inception to February 1, 2026. The search strategies combined subject headings, where available, and free-text terms related to stroke, aphasia/language impairment, and inhibitory control/executive control. No language restrictions were applied. Search strategies were adapted to the syntax of each database.

# Pubmed

**Coverage:**database inception to February 1, 2026
**Language restrictions:** none

("Stroke"[Mesh] OR "Stroke"[Title/Abstract] OR "Post-stroke"[Title/Abstract] OR "Cerebrovascular accident"[Title/Abstract] OR "CVA"[Title/Abstract] OR "Brain infarction"[Title/Abstract])

AND

("Aphasia"[Mesh] OR "Aphasia"[Title/Abstract] OR "Dysphasia"[Title/Abstract] OR "Anomia"[Title/Abstract] OR "Language impairment"[Title/Abstract] OR "Language disorder"[Title/Abstract])

AND

("Inhibition"[Title/Abstract] OR "Inhibitory control"[Title/Abstract] OR "Executive function"[Title/Abstract] OR "Cognitive control"[Title/Abstract] OR "Attention"[Title/Abstract] OR "Interference control"[Title/Abstract] OR "Stroop"[Title/Abstract] OR "Flanker"[Title/Abstract] OR "Simon task"[Title/Abstract] OR "Go/no-go"[Title/Abstract] OR "Stop-signal"[Title/Abstract] OR "Hayling"[Title/Abstract])

# Web of Science

**Coverage:**database inception to February 1, 2026
**Language restrictions:** none

TS=(("Aphasia" OR "Dysphasia" OR "Anomia" OR "Language disorder*" OR "Language impairment*")

AND

("Stroke" OR "Post-stroke" OR "Cerebrovascular accident" OR "CVA" OR "Brain infarction" OR "Brain hemorrhage")

AND

("Inhibition" OR "Inhibitory control" OR "Response inhibition" OR "Interference control" OR "Executive function*" OR "Executive control" OR "Cognitive control" OR "Selective attention" OR "Stroop" OR "Flanker" OR "Simon task" OR "Go/no-go" OR "Stop-signal" OR "Hayling"))

# Embase

**Coverage:**database inception to February 1, 2026
**Language restrictions:** none

('aphasia':ti,ab,kw OR 'dysphasia':ti,ab,kw OR 'anomia':ti,ab,kw)

AND

('stroke':ti,ab,kw OR 'cerebrovascular accident':ti,ab,kw OR 'cva':ti,ab,kw)

AND

('inhibition':ti,ab,kw OR 'inhibitory control':ti,ab,kw OR 'executive function':ti,ab,kw OR 'cognitive control':ti,ab,kw OR 'stroop':ti,ab,kw OR 'flanker':ti,ab,kw OR 'simon task':ti,ab,kw OR 'go no go':ti,ab,kw)

# Cochrane

**Coverage:**database inception to February 1, 2026
**Language restrictions:** none

([mh "Aphasia"] OR aphasia*:ti,ab,kw OR dysphasia*:ti,ab,kw OR anomia*:ti,ab,kw OR "language disorder*":ti,ab,kw OR "language impairment*":ti,ab,kw)

AND

([mh "Stroke"] OR [mh "Cerebrovascular Disorders"] OR stroke:ti,ab,kw OR "post-stroke":ti,ab,kw OR "cerebrovascular accident":ti,ab,kw OR CVA:ti,ab,kw)

AND

([mh "Executive Function"] OR [mh "Inhibition, Psychological"] OR [mh "Attention"] OR inhibit*:ti,ab,kw OR "executive function":ti,ab,kw OR "cognitive control":ti,ab,kw OR "selective attention":ti,ab,kw OR stroop:ti,ab,kw OR flanker:ti,ab,kw OR "simon task":ti,ab,kw OR "go no go":ti,ab,kw OR "stop signal":ti,ab,kw OR hayling:ti,ab,kw)

# CNKI

**Coverage:**database inception to February 1, 2026
**Language restrictions:** non

主题 = ('失语' OR '失语症' OR '言语障碍' OR '语言障碍')

AND

主题 = ('脑卒中' OR '中风' OR '脑梗' OR '脑梗死' OR '脑出血' OR '脑血管意外')

AND

主题 = ('抑制' OR '抑制控制' OR '执行功能' OR '认知控制' OR 'Stroop' OR '斯特鲁普' OR 'Flanker' OR 'Simon' OR 'Go/No-Go' OR '停止信号')

# Wanfang Data

**Coverage:**database inception to February 1, 2026
**Language restrictions:** non

主题: ("失语" OR "失语症" OR "言语障碍" OR "语言障碍")

AND

主题: ("脑卒中" OR "中风" OR "脑梗死" OR "脑梗" OR "脑出血" OR "脑血管意外")

AND

主题: ("抑制" OR "抑制控制" OR "执行功能" OR "认知控制" OR "Stroop" OR "斯特鲁普" OR "Flanker" OR "Simon" OR "Go/No-Go" OR "停止信号")
