# Supplementary figures and images for: The association between cognitive inhibitory control and language performance in post-stroke aphasia: a systematic review and meta-analysis
Source: Front Psychol. 2026 May 26;17:1846757. doi: 10.3389/fpsyg.2026.1846757 (PMC13246680; doi:10.3389/fpsyg.2026.1846757)

***SM3 Between-Group Difference Test:***

***Interference Control and Response Inhibition***


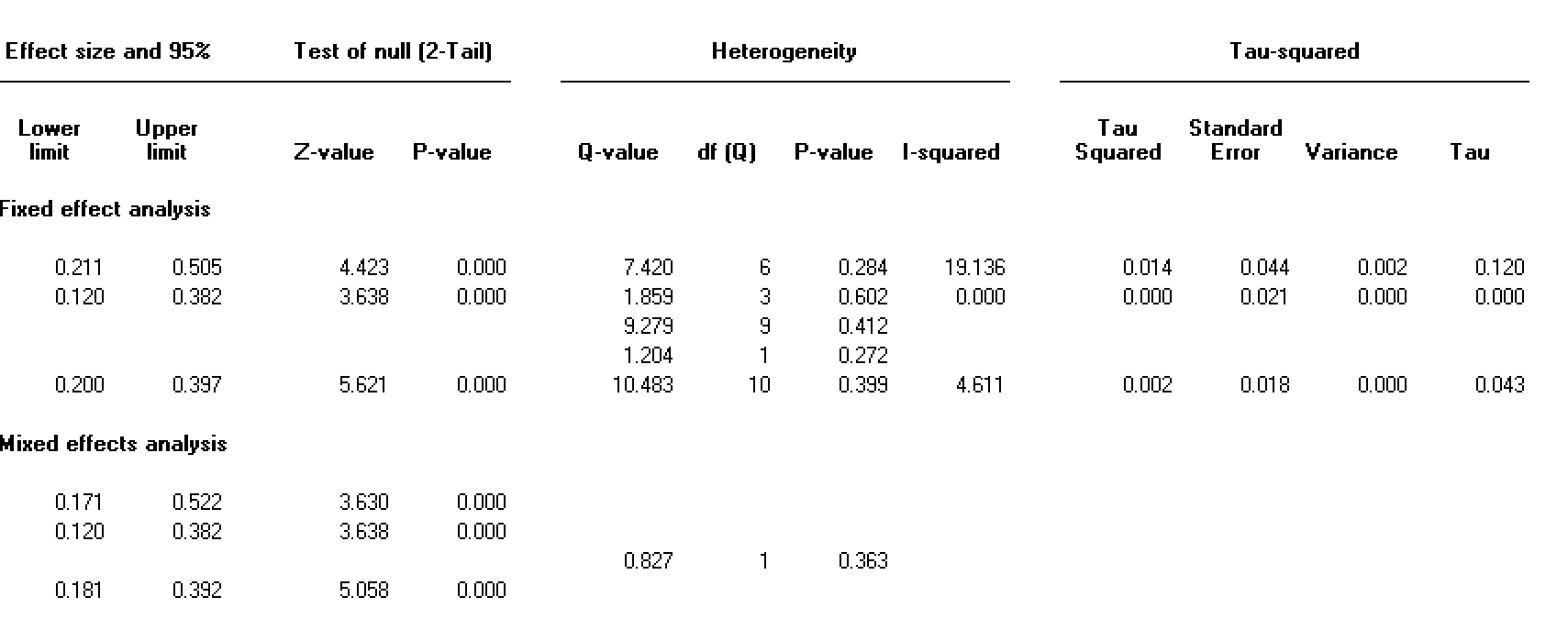

Supplement: Supplementary file 3 [file Supplementary_File_3.docx]
